# Amniotic and cervical fluids progranulin levels in pregnancies complicated by spontaneous preterm delivery with respect to intra-amniotic complications—a retrospective cohort study

**DOI:** 10.1038/s41598-025-05887-0

**Published:** 2025-07-11

**Authors:** Ondrej Soucek, Marian Kacerovsky, Ivana Musilova, Rudolf Kukla, Radka Bolehovska, Pavel Bostik, Bo Jacobsson, Ctirad Andrys

**Affiliations:** 1 Department of Clinical Immunology and Allergology University Hospital Hradec Kralove, Faculty of Medicine in Hradec Kralove, Charles University, Prague, Czech Republic; 2 Biomedical Research Center, University Hospital Hradec Kralove, Sokolska 581, Hradec Kralove, 500 05 Czech Republic; 3 Department of Obstetrics and Gynecology, University Hospital Olomouc, Olomouc, Czech Republic; 4 Institute of Clinical Microbiology, Faculty of Medicine in Hradec Kralove, Charles University, Prague, Czech Republic; 5 Institute of Clinical Microbiology, University Hospital, Hradec Kralove, Czech Republic; 6 Department of Obstetrics and Gynecology, Institute of Clinical Science, Sahlgrenska Academy, University of Gothenburg, Gothenburg, Sweden; 7 Region Västra Götaland, Department of Obstetrics and Gynecology, Sahlgrenska University Hospital, Gothenburg, Sweden; 8 Department of Genetics and Bioinformatics, Domain of Health Data and Digitalisation, Institute of Public Health, Oslo, Norway

**Keywords:** Amniotic fluid, Invasive sampling, Intra-amniotic inflammation, Microbial invasion of the amniotic cavity, Non-invasive sampling, Preterm delivery, Biomarkers, Preclinical research

## Abstract

The main aim of the study was to determine progranulin levels in amniotic and cervical fluid samples from pregnancies complicated by preterm prelabor rupture of membranes (PPROM) or preterm labor with intact membranes (PTL), with concomitant microbial invasion of the amniotic cavity and/or intra-amniotic inflammation. A total of 104 and 108 women with PPROM and PTL, respectively, were included. Paired amniotic and cervical fluid samples were obtained using transabdominal amniocentesis and Dacron polyester swabs, respectively. Progranulin levels were assessed with an enzyme-linked immunosorbent assay. Women with PPROM and PTL were divided into subgroups based on microbial invasion of the amniotic cavity and/or intra-amniotic inflammation. Differences in progranulin levels among the PPROM and PTL subgroups were found in amniotic fluid: (a) PPROM: intra-amniotic infection: 51.8 pg/mL, sterile intra-amniotic inflammation: 52.8 pg/mL, colonization: 36.4 pg/mL, and negative amniotic fluid: 35.0 pg/mL; *p* < 0.0001; (b) PTL: intra-amniotic infection: 75.3 pg/mL, sterile intra-amniotic inflammation: 54.0 pg/mL, and negative amniotic fluid: 39.1 pg/mL; *p* < 0.0001. The corresponding differences were not found in cervical fluid: (a) PPROM: *p* = 0.14; (b) PTL: *p* = 0.53. In conclusion, amniotic fluid progranulin levels increased in PPROM and PTL cases with concomitant intra-amniotic inflammation, regardless of whether microbial invasion of the amniotic cavity was present or absent.

**Supplementary Information:**

The online version contains supplementary material available at 10.1038/s41598-025-05887-0.

## Introduction

If delivery occurs before 37 gestational weeks, it is defined as preterm delivery, a condition affecting about 10% of pregnancies. The mode of labor onset divides preterm delivery into two categories: (a) iatrogenic, i.e. initiated by a physician’s decision, and (b) spontaneous^1,2^. The latter has two subtypes: (i) preterm prelabor rupture of membranes (PPROM), with amniotic fluid discharge in the absence of uterine contractions; and (ii) preterm labor (PTL), i.e. regular uterine contractions with intact membranes, leading to altered cervical status^1,2^. Both PPROM and PTL are often complicated by the presence of microorganisms in amniotic fluid (microbial invasion of the amniotic cavity)^3,4^, which can trigger a host innate immune defense reaction, generating an inflammatory process cascade and, ultimately, increasing inflammatory mediators and neutrophils levels in the amniotic fluid (intra-amniotic inflammation)^4–6^.

Neutrophils mainly contribute to the defense against microorganisms in three ways: (a) phagocytosis of microorganisms, followed by their intra-cellular degradation^7^; (b) formation of neutrophil extracellular traps that can catch and kill microorganisms^7,8^; and (c) degranulation with the discharge of antimicrobial factors, such as neutrophil serine proteases (e.g. cathepsin G, neutrophil elastase), which can kill microorganisms, digest tissue and affect inflammatory response^7,9^. These processes are tightly orchestrated and thoroughly regulated^7^. One of the many controlling and regulatory mediators in this process is progranulin, which controls extravasation of neutrophils from blood vessels^7,10^.

Progranulin, a multifunctional glycoprotein involved in inflammation, wound healing, cell growth, embryogenesis, tumorigenesis and insulin resistance^11^, was identified in 1990^12^. After its secretion, the full-length protein is cleaved into individual granulin peptides^13,14^. Progranulin and granulins are biologically active; progranulin plays an anti-inflammatory role, whereas granulins appear to have an inflammatory function^11^.

When intra-amniotic inflammation has been investigated in cases of PPROM and/or PTL, amniotic fluid levels of some of these serine proteases (cathepsin-G, neutrophil elastase) have been found to be elevated^15–17^. Since progranulin can be inactivated by these proteases^7,10^, our hypothesis was thus that progranulin levels in amniotic fluid might also be altered in cases with intra-amniotic inflammation. Moreover, progranulin cervical fluid levels may also be affected because: (a) the cervix is located the closest to the amniotic cavity; (b) levels of interleukin (IL) -6 in cervical fluid reflect the presence of intra-amniotic complications^18,19^; and (c) cervical fluid progranulin participates in the cervical remodeling process^20^.

The main goals of this retrospective study were thus to measure progranulin levels in amniotic and cervical fluid samples from women with PPROM and women with PTL, and to investigate the association between progranulin levels and the presence of microbial invasion of the amniotic cavity and/or intra-amniotic inflammation.

## Results

This study included 108 women with PTL and 104 women with PPROM. Among the women with PTL, 17% (18/108) had intra-amniotic infection, 32% (34/108) had sterile intra-amniotic inflammation and 52% (56/108) had negative amniotic fluid. There were no cases of colonization in this group. When it came to the PPROM cases, 14% (15/104) had intra-amniotic infection, 11% (11/104) had sterile intra-amniotic inflammation, 19% (20/104) had colonization and 56% (58/104) had negative amniotic fluid.

The respective demographic and clinical data in the PPROM and PTL groups are shown in Tables 1 and 2, respectively, together with data concerning presence or absence of intra-amniotic inflammation and/or microbial invasion of the amniotic cavity. Table 3 presents the microorganisms detected in the respective group’s amniotic fluid.

**Table 1:** Demographic and clinical characteristics of women with preterm prelabor rupture of membranes with intra-amniotic infection, sterile intra-amniotic inflammation, colonization of the amniotic cavity, and negative amniotic fluid.

Characteristic	Intra-amniotic infection (*n* = 15)	Sterile intra-amniotic inflammation (*n* = 11)	Colonization of the amniotic cavity (*n* = 20)	Negative amniotic fluid (*n* = 58)	*p*-value
Maternal age [years, median (IQR)]	32 (30.36)	36 (29–41)	30 (27–33)	31 (27–35)	0.19
Primiparous [number (%)]	8 (53%)	4 (45%)	7 (35%)	31 (54%)	0.42
Smoking [number (%)]	3 (20%)	1 (9%)	5 (25%)	3 (5%)	0.07
Pre-pregnancy body mass index [kg/m^2^, median (IQR)]	25.9 (20.7–30.1)	26.7 (21.4–28.9)	24.0 (19.5–27.5)	24.5 (21.2–29.2)	0.67
Gestational age at sampling [weeks + days, median (IQR)]	29 + 3 (26 + 6–30 + 5)	30 + 0 (24 + 0–33 + 4)	33 + 2 (32 + 0–35 + 3)	33 + 2 (30 + 1–35 + 0)	**< 0.0001**
Gestational age at delivery [weeks + days, median (IQR)]	30 + 4 (27 + 4–32 + 3)	32 + 4 (27 + 1–34 + 0)	34 + 1 (33 + 0–35 + 3)	34 + 1 (32 + 0–35 + 5)	**< 0.0001**
Latency from PPROM to amniocentesis [hours, median (IQR)]	5 (3–21)	10 (5–16)	4 (2–6)	4 (2–10)	0.11
Latency from PPROM to delivery [hours, median (IQR)]	141 (60–305)	227 (36–536)	42 (12–106)	142 (26–312)	**0.02**
Amniotic fluid IL-6 levels at admission [ng/mL, median (IQR)]	13.0 (5.7–33.3)	5.6 (3.5–10.5)	1.1 (0.8–1.6)	0.8 (0.4–1.4)	**< 0.0001**
CRP levels at admission [mg/L, median (IQR)]	10.7 (3.9–21.9)	6.5 (4.7–12.6)	4.6 (2.8–8.1)	4.7 (2.3–8.5)	0.12
WBC count at admission [x10^9^ L, median (IQR)]	11.4 (9.6–14.3)	11.8 (10.7–15.2)	11.4 (9.8–14.7)	12.3 (10.4–14.3)	0.92
Administration of corticosteroids [number (%)]	15 (100%)	10 (91%)	14 (70%)	42 (72%)	0.07
Administration of antibiotics [number (%)]	15 (100%)	10 (91%)	19 (95%)	57 (98%)	0.47
Spontaneous vaginal delivery [number (%)]	11 (73%)	6 (55%)	13 (65%)	43 (74%)	0.56
Cesarean section [number (%)]	4 (27%)	5 (45%)	7 (35%)	15 (26%)	0.56
Birth weight [grams, median (IQR)]	1500 (1100–1890)	1960 (880–2130)	2150 (1685–2445)	2125 (1743–2630)	**0.002**
Apgar score < 7; 5 min [number (%)]	2 (13%)	1 (9%)	1 (5%)	1 (2%)	0.26
Apgar score < 7; 10 min [number (%)]	1 (7%)	1 (9%)	1 (5%)	1 (2%)	0.59

Continuous variables, presented as median (interquartile range), were compared using a nonparametric Kruskal-Wallis test. Categorical variables, presented as number (%), were compared using chi-square test. Statistically significant results are marked in bold.

*CRP* C-reactive protein, *IL* interleukin, *IQR* interquartile range, *WBC* white blood cells.

**Table 2:** Demographic and clinical characteristics of women with spontaneous preterm labor with intra-amniotic infection, sterile intra-amniotic inflammation, and negative amniotic fluid.

Characteristic	Intra-amniotic infection (*n* = 18)	Sterile intra-amniotic inflammation (*n* = 34)	Negative amniotic fluid (*n* = 56)	*P*-value
Maternal age [years, median (IQR)]	28 (28–33)	28 (26–32)	29 (23–33)	0.78
Primiparous [number (%)]	8 (44%)	23 (68%)	33 (59%)	0.27
Smoking [number (%)]	4 (22%)	7 (21%)	7 (13%)	0.48
Pre-pregnancy body mass index [kg/m^2^, median (IQR)]	26.4 (23.3–31.1)	23.9 (20.9–30.1)	23.4 (19.8–27.8)	0.08
Gestational age at sampling [weeks + days, median (IQR)]	27 + 4 (24 + 0–30 + 2)	26 + 5 (24 + 3–30 + 5)	31 + 6 (29 + 6–33 + 6)	**< 0.0001**
Gestational age at delivery [weeks + days, median (IQR)]	28 + 1 (24 + 2–31 + 1)	27 + 4 (25 + 1–32 + 0)	34 + 6 (31 + 5–37 + 4)	**< 0.0001**
Amniotic fluid IL-6 levels at admission [ng/mL, median (IQR)]	50.0 (33.9–50.0)	7.1 (4.6–24.5)	7.4 (4.0-1.6)	**< 0.0001**
CRP levels at admission [mg/L, median (IQR)]	33.8 (9.8–50.4)	10.8 (6.8–17.5)	5.5 (2.3–9.7)	**< 0.0001**
WBC count at admission [x10^9^ L, median (IQR)]	14.7 (11.5–19.3)	14.7 (11.3–16.8)	11.4 (9.9–15.2)	**0.02**
Administration of corticosteroids [number (%)]	18 (100%)	34 (100%)	56 (100%)	-
Administration of antibiotics [number (%)]	17 (94%)	32 (94%)	0 (0%)	**< 0.0001**
Interval between admission and deliver [days, median (IQR)]	0 (0–3)	2 (0–4)	4 (1–43)	**0.008**
Spontaneous vaginal delivery [number (%)]	13 (72%)	29 (85%)	43 (77%)	0.48
Cesarean section [number (%)]	5 (28%)	5 (15%)	13 (23%)	0.48
Birth weight [grams, median (IQR)]	1020 (705–1630)	1055 (770–2740)	2195 (1798–3038)	**< 0.0001**
Apgar score < 7; 5 min [number (%)]	7 (39%)	11 (32%)	3 (5%)	**0.0005**
Apgar score < 7; 10 min [number (%)]	6 (33%)	7 (21%)	1 (2%)	**0.0006**

Continuous variables, presented as median (interquartile range), were compared using a nonparametric Kruskal-Wallis test. Categorical variables, presented as number (%), were compared using chi-square test. Statistically significant results are marked in bold.

*CRP* C-reactive protein, *IL* interleukin, *IQR* interquartile range, *WBC* white blood cells.

**Table 3:** The microbial species identified in the amniotic fluid of women with preterm prelabor rupture of membranes and with spontaneous preterm labor with intact membranes.

Preterm prelabor rupture of membranes	Preterm labor with intact membranes
*Corynebacterium tuberculostearicum + Staphylococcus epidermidis + Dermatobacter hominis (n* = 1)	*Ureaplasma* spp. + *Capnocytophaga ochracea + Fusobacterium nucleatum* (*n* = 1)
*Lactobacillus gasseri + Gardnerella vaginalis + Prevotella bivia* (*n* = 1)	*Fusobacterium nucleatum + Leptotrichia trevisanii* (*n* = 1)
*Ureaplasma* spp. + *Gardnerella vaginalis + Prevotella disiens* (*n* = 1)	*Klebsiella pneumoniae + Streptococcus anginosus* (*n* = 1)
*Ureaplasma* spp. + *Sneathia sanguinises + Lactobacillus iners* (*n* = 1)	*Lactobacillus plantum + Gardnerella vaginalis* (*n* = 1)
*Ureaplasma* spp. + *Mycoplasma hominis* (*n* = 2)	*Ureaplasma* spp. (*n* = 5)
*Ureaplasma* spp. + *Streptococcus oralis* (*n* = 1)	*Fusobacterium nucleatum* (*n* = 2)
*Ureaplasma* spp. (*n* = 13)	*Burkholderia cepacia* (*n* = 1)
*Escherichia coli* (*n* = 2)	*Hemophilus influenzae* (*n* = 1)
*Mycoplasma hominis* (*n* = 2)	*Lactococcus lactis* (*n* = 1)
*Clostridium perfringens* (*n* = 1)	*Sneathia sanquinegens* (*n* = 1)
*Gardnerella vaginalis* (*n* = 1)	*Staphylococcus epidermidis* (*n* = 1)
*Moraxella osloensis* (*n* = 1)	Non-identifiable bacteria by sequencing (*n* = 2)
*Peptoniphilus indolicus* (*n* = 1)	
*Peptoniphilus* spp. (*n* = 1)	
*Peptostreptococcus stomatis* (*n* = 1)	
*Sneathia sanguinegens* (*n* = 1)	
*Streptococcus agalactiae* (*n* = 1)	
*Streptococcus hominis* (*n* = 1)	
*Streptococcus oralis* (*n* = 1)	
*Streptococcus sanquinis* (*n* = 1)	

Progranulin levels were measurable in all amniotic and cervical fluid samples in both groups.

### Amniotic fluid progranulin levels in spontaneous PTD subtype groups

#### (a) PPROM

**Fig. 1:**
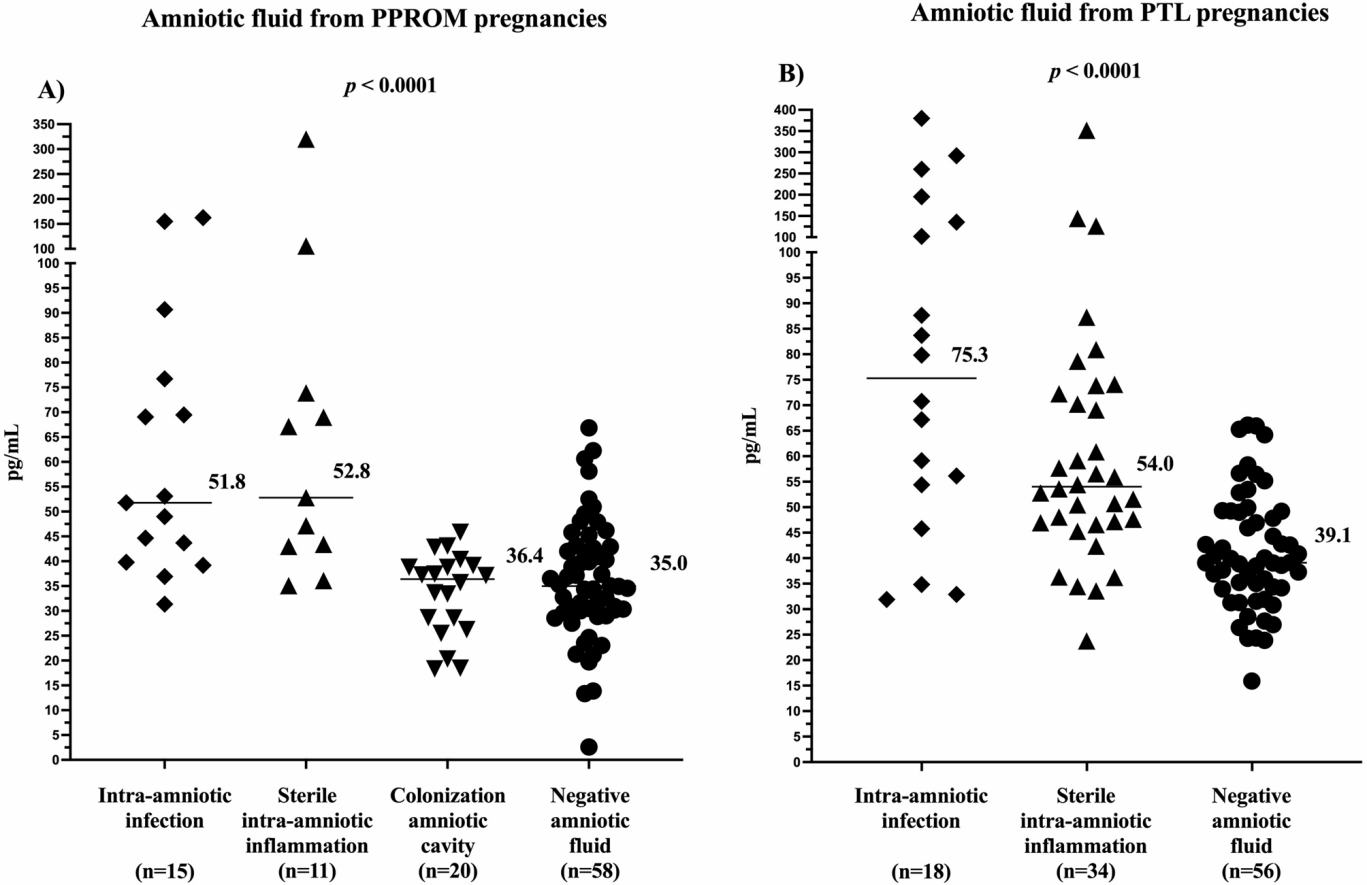
Amniotic fluid progranulin levels in the PPROM (**A**) and PTL (**B**) subgroups. *PPROM* preterm pre-labor rupture of membranes, *PTL* preterm labor with intact membranes.

There was a correlation between progranulin and IL-6 levels in amniotic fluid (rho = 0.62, *p* < 0.0001). Women with intra-amniotic infection, sterile intra-amniotic inflammation, colonization and negative amniotic fluid had differing progranulin levels, both in the crude analysis (infection: median 51.8 pg/mL, IQR 39.8–76.7; sterile: median 52.8 pg/mL, IQR 43.0-52.8; colonization: median 36.4 pg/mL, IQR 26.8–36.4; negative 35.0 pg/mL, IQR 29.9–42.7; *p* < 0.0001; Fig. 1A) and after adjustment for gestational age *(p =* 0.002). Progranulin levels in the intra-amniotic infection and sterile intra-amniotic inflammation subgroups exceeded levels in the colonization and negative amniotic fluid (Table 4). No differences in amniotic fluid progranulin levels were found between women with intra-amniotic infection or sterile intra-amniotic inflammation and those with colonization or negative amniotic fluid (Table 4).

**Table 4:** Comparisons of levels of progranulin among the subsets of preterm prelabor rupture of membranes (a) and spontaneous preterm labor (b) groups divided based on the presence or absence of microbial invasion of the amniotic fluid and intra-amniotic inflammation.

	Intra-amniotic infection	Sterile intra-amniotic inflammation	Colonization of the amniotic cavity	Negative amniotic fluid
(a)				
Intra-amniotic infection	–	*p* = 0.96	***p*** **< 0.0001/*p*** = 0.0005^*^	***p*** **< 0.0001/*p*** = 0.0004^*^
Sterile intra-amniotic inflammation	*p* = 0.96	–	***p*** **= 0.0002/*p*** = 0.001^*^	***p*** **< 0.0001/*p*** = 0.002^*^
Colonization of the amniotic cavity	***p*** **< 0.0001/*p*** = 0.0005^*^	***p*** **= 0.0002/*p*** = 0.001^*^	–	*p* = 0.45
Negative amniotic fluid	***p*** **< 0.0001/*p*** = 0.0004^*^	***p*** **< 0.0001/*p*** = 0.002^*^	*p* = 0.45	–
(b)				
Intra-amniotic infection	–	*p* = 0.07		***p*** **< 0.0001/*p*** < 0.0001^*^
Sterile intra-amniotic inflammation	*p* = 0.07	–		***p*** **< 0.0001/*p <*** 0.0001^*^
Negative amniotic fluid	***p*** **< 0.0001/*p*** < 0.0001^*^	***p*** **< 0.0001/*p*** < 0.0001^*^		–

Variables were compared using the Mann-Whitney *U* test. Statistically significant results are marked in bold.

*Adjusted for gestational age at sampling.

#### (b) PTL

There was a correlation between amniotic fluid progranulin and IL-6 levels (rho = 0.57, *p* < 0.0001). A difference in progranulin levels was revealed in the intra-amniotic infection, sterile intra-amniotic inflammation and negative amniotic fluid (infection: median 75.3 pg/mL, IQR 52.3-150.3; sterile: median 54.0 pg/mL, IQR: 46.9–72.7; negative: median 39.1 pg/mL, IQR: 34.0-49.2; *p* < 0.0001; Fig. 1B) subgroups, both in the crude analysis (*p* < 0.0001) and after adjustment for gestational age (*p* = 0.001). Women with intra-amniotic infection and sterile intra-amniotic inflammation had higher amniotic fluid progranulin levels than those with negative amniotic fluid (Table 4). No difference in amniotic fluid progranulin levels was found between those with intra-amniotic infection and those with sterile intra-amniotic inflammation (Table 4).

### Cervical fluid progranulin levels in spontaneous PTD subtype groups

#### (a) PPROM

**Fig. 2:**
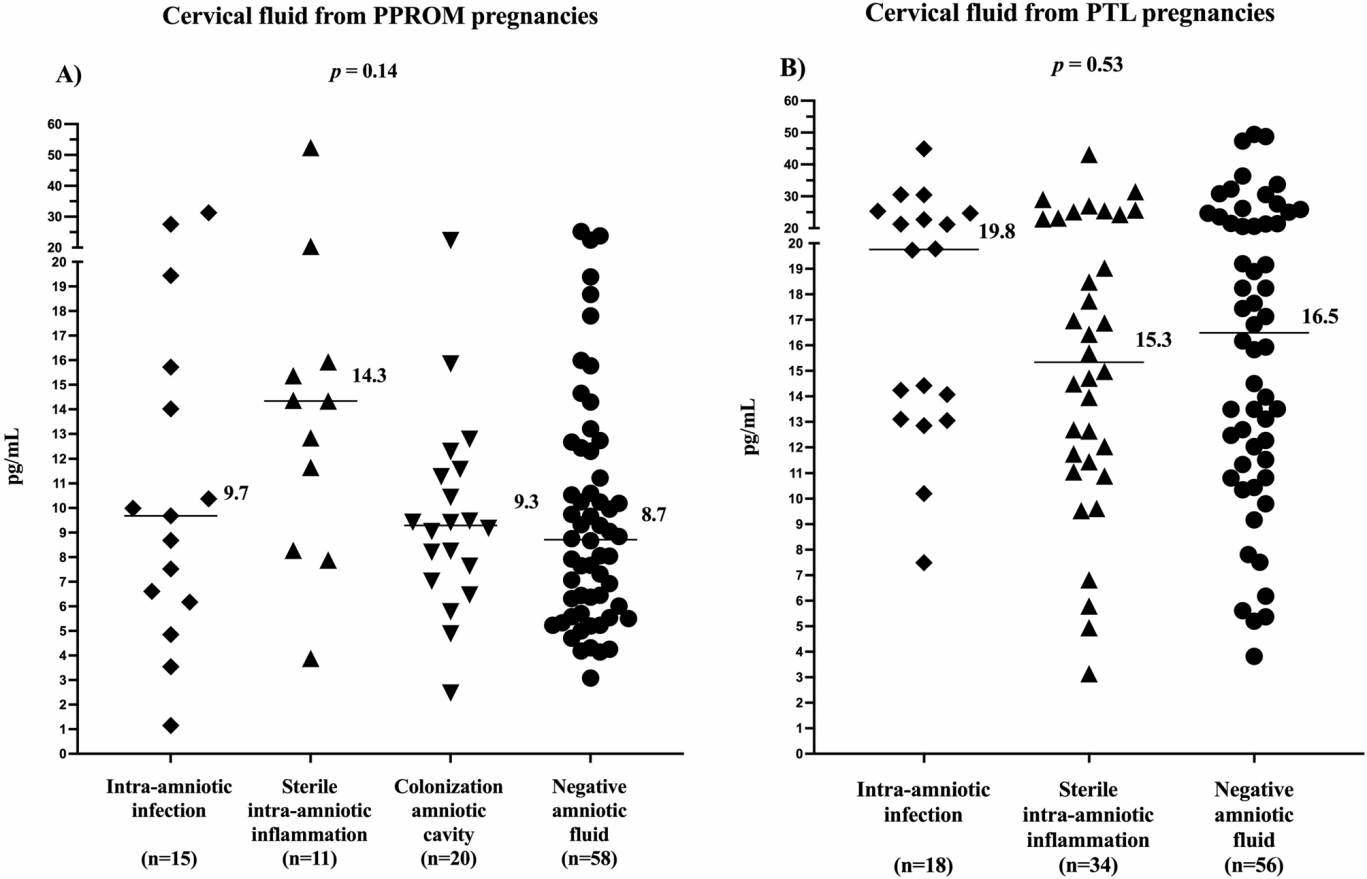
Cervical fluid progranulin levels in the PPROM (**A**) and PTL (**B**) subgroups. *PPROM* preterm pre-labor rupture of membranes, *PTL* preterm labor with intact membranes.

**Fig. 3:**
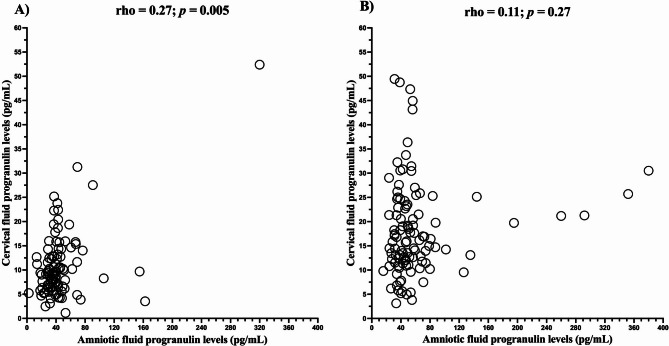
Correlations between amniotic and cervical fluid progranulin levels in the PPROM (**A**) and PTL (**B**) subgroups. *PPROM* preterm pre-labor rupture of membranes, *PTL* preterm labor with intact membranes.

The subgroups exhibited no differences (infection: median 9.7 pg/mL, IQR 6.2–15.7; sterile: median 14.3 pg/mL, IQR 8.3–15.9; colonization: median 9.3 pg/mL, IQR 7.2–11.5; negative: median 8.7 pg/mL, IQR 5.7–8.7; *p* = 0.14; Fig. 2A) in cervical fluid progranulin levels. The progranulin levels cervical fluid correlated weakly with progranulin levels in amniotic fluid (*p* = 0.27; *p* = 0.005; Fig. 3A). In addition, there was a weak positive correlation between cervical fluid progranulin and amniotic fluid IL-6 levels (rho = 0.21, *p* = 0.03).

#### (b) PTL

Any differences in progranulin levels in cervical fluid were revealed among the subgroups (infection: median 19.8 pg/mL, IQR 13.1–24.8; sterile: median 15.3 pg/mL, IQR 11.4–23.5; negative: median 16.5 pg/mL, IQR 11.4–23.0; *p* = 0.53; Fig. 2B). In contrast to the PPROM group, no correlations were found between cervical fluid and amniotic fluid progranulin levels (*p* = 0.11; *p* = 0.27; Fig. 3B), and cervical fluid progranulin and IL-6 levels (rho = 0.10, *p* = 0.28).

## Discussion

### Principal findings

(1) The hypothesis that progranulin levels in amniotic fluid might be altered in concomitant presence of intra-amniotic inflammation was proven since its levels were elevated in PPROM and PTL pregnancies with concomitant intra-amniotic inflammation, regardless of whether or not there was microbial invasion of the amniotic cavity; 2) the hypothesis that progranulin levels in cervical fluid might be altered in concomitant presence of intra-amniotic inflammation was not proven since cervical fluid progranulin levels were unaffected, both in PTL and PPROM pregnancies, by concomitant microbial invasion of the amniotic cavity and/or intra-amniotic inflammation.

### Meaning of the study

Finding progranulin in second-trimester amniotic fluid samples was first reported in 2011 by Stubert et al.^21^. This finding was confirmed in this study, where progranulin was present in all amniotic fluid samples from pregnancies affected by PPROM or PTL. When it came to intra-amniotic complications common to these two conditions, amniotic fluid progranulin levels were higher in cases of intra-amniotic inflammation, regardless of whether microorganisms were found in the amniotic fluid. This concurs with the following findings in previous reports on amniotic fluid levels of serine proteases (which can inactivate progranulin): (a) if either PTL or PPROM is complicated by microbial invasion of the amniotic cavity, higher neutrophil elastase levels are found than in cases with no microorganisms in amniotic fluid^15^; and (b) regardless of whether microbial invasion of the amniotic cavity is detected, amniotic fluid cathepsin-G levels are higher in cases with PPROM and intra-amniotic inflammation than in those with PPROM but no intra-amniotic inflammation^22^. In summary, amniotic fluid levels of progranulin and serine proteases undergo similar alterations if there is concomitant intra-amniotic inflammation.

In pregnancy, progranulin is produced by the cervix, where it contributes to the process of cervix remodeling^20^. In a mouse model of pregnancy, production of progranulin was found in the cervical stroma but not in the cervical epithelium^20^. This study confirmed the observation in other studies that the cervix produces progranulin, which was measurable in all samples from PPROM and PTL cases. Cervical fluid progranulin levels were 4-fold and 3-fold lower, respectively, than the corresponding levels in amniotic fluid from PPROM and PTL pregnancies. It must, however, be kept in mind that the cervical fluid samples in this study were diluted (cervical fluid was obtained from a Dacron swab placed in the cervical canal for 20 s to achieve saturation; the fluid was subsequently diluted in 1.5 mL of buffer).

In this study, no association between intra-amniotic complications and cervical niche progranulin levels was found either in PTL or even in PPROM, where the cervical fluid might have been contaminated by leakage of amniotic fluid. This finding concurred with results of our previous study of cathepsin-G levels in women with PPROM^23^. Findings in a previous study on cervical fluid progranulin levels indicate that associations are stronger between cervical fluid progranulin level changes and cervical length than the corresponding associations with intra-amniotic complications^20^. In that study, cervical length < 35 mm was associated with lower cervical fluid progranulin levels than cervical length exceeding 35 mm^20^.

### Strengths and limitations of the study

The relatively large cohort of paired cervical and amniotic fluid samples from both subtypes of spontaneous preterm delivery is the strength of this study. On the other hand, there were also some limitations. Progranulin levels were not analyzed in other body fluids (e.g. umbilical cord or maternal blood). Moreover, sources of amniotic and cervical fluid progranulin were not investigated. Amniotic fluid leakage through the cervix in case of PPROM is an inevitable confounder in cervical fluid sampling, another limitation when studying this subtype of spontaneous preterm delivery.

## Conclusion

Amniotic fluid progranulin levels increased in PPROM and PTL cases with concomitant intra-amniotic inflammation, regardless of whether intra-amniotic inflammation was present or absent.

## Materials and methods

This retrospective cohort study was conducted at the Department of Obstetrics and Gynecology at the University Hospital Hradec Kralove in the Czech Republic. Included participants were pregnant women admitted to the department between March 1, 2019, and August 31, 2022. Inclusion criteria were: (a) singleton pregnancy; (b) maternal age ≥ 18 years; (c) PPROM at 24 + 0–36 + 6 gestational weeks; (d) PTL at 22 + 0–34 + 6 gestational weeks; (e) transabdominal amniocentesis performed at admission to assess intra-amniotic inflammation; and (f) cervical fluid sample available. Exclusion criteria were: (a) general medical or obstetric complications, such as fetal growth restriction, pre-gestational or gestational diabetes, chronic or gestational hypertension and preeclampsia; (b) chromosomal or structural fetal abnormality; (c) signs of fetal hypoxia; and (d) significant vaginal bleeding.

Fetal biometry in the first trimester was the basis for determining gestational age. PPROM diagnosis was based on sterile speculum examination to detect pooling of amniotic fluid in the vaginal posterior fornix. If PPROM diagnosis was uncertain, amniotic fluid leakage was confirmed by testing vaginal fluid for insulin-like growth factor-binding proteins (Actim PROM test; Medix Biochemica, Kauniainen, Finland)^24,25^.

Regular uterine contractions (≥ two contractions per 10 min), plus cervical length < 15 mm or cervical length 15–30 mm with a positive PartoSure test (Parsagen Diagnostics Inc., Boston, MA, USA), were the diagnostic criteria for PTL^24–26^. Cervical length was measured by transvaginal ultrasound.

Women with PPROM were administered intravenous (IV) antibiotics: benzylpenicillin IV (or clindamycin IV in case of penicillin allergy) for those without intra-amniotic inflammation and clarithromycin in cases with intra-amniotic inflammation. Treatment continued for seven days unless delivery occurred. Women with PPROM and intra-amniotic infection at gestational age > 28 + 0 weeks underwent induction of labor or elective cesarean section within 72 h of admission, while the remaining women with PPROM were managed expectantly^24^.

Atosiban IV was administered for 48 h as tocolytic treatment of women with PTL. Additional treatment with clarithromycin IV, for seven days unless delivery occurred earlier, was given to those who also had intra-amniotic inflammation^24^.

Women with PPROM and PTL at gestational age < 35 + 0 weeks were given intramuscular betamethasone, according to regimen, aimed at reducing neonatal morbidity and mortality via accelerated fetal lung maturation.

During active labor, women with PPROM or PTL with positive vaginal-rectal swabs for group B *Streptococcus*, or with unclear group B *Streptococcus* status, were given benzylpenicillin IV (or clindamycin, in case of penicillin allergy)^24^.

All women were Caucasian and provided written informed consent prior to sampling for this study. Ethical approval for collection of body fluids for research, was granted by the Institutional Review Board of the University Hospital Hradec Kralove (February 2019, No. 201902 S16P). This retrospective study was approved by the Institutional Review Board of University Hospital Hradec Kralove (September 2023, No. 202309 P01). Prior to analysis, which began 22 September 2023, all data were fully anonymized. Information enabling identification of individual participants during and after data collection was accessible to the authors. All sampling and analyses were performed in accordance with the relevant guidelines and regulations.

### Sampling of cervical and amniotic fluid

Paired cervical and amniotic fluid samples were collected at admission from all participants, after which antibiotics, tocolytics and/or corticosteroids were given. Detailed description of both sampling techniques can be found in our previous papers^18,22,27,28^.

### Amniotic fluid IL-6 levels

A Cobas e602 immunoanalyzer, part of the Cobas 8000 platform (Roche Diagnostics, Basel, Switzerland), was used to assess IL-6 levels^29^.

### Detection of Ureaplasma spp., M. hominis, C. trachomatis and other bacteria in amniotic fluid

The commercial AmpliSens^®^ *C. trachomatis*/*Ureaplasma*/*M. hominis*-FRT kit (Federal State Institution of Science, Central Research Institute of Epidemiology, Moscow, Russia) was used to detect *Ureaplasma* spp., *M. hominis* and *C. trachomatis* DNA. A previous publication of ours^24^ describes in detail the detection of other bacteria in amniotic fluid by sequencing the 16 S rRNA gene.

### Aerobic and anaerobic cultures of amniotic fluid

Our above-mentioned paper^24^ describes aerobic and anaerobic cultures of amniotic fluid.

### Clinical definitions

Microbial invasion of the amniotic cavity was identified based on: (a) positive PCR for *Ureaplasma* spp., *M. hominis* or *C. trachomatis*; or (b) positive PCR for a combination of these species; and/or (c) positivity for the 16 S rRNA gene; and/or (d) positive aerobic or anaerobic amniotic fluid culture^24^. Intra-amniotic inflammation was defined as amniotic fluid IL-6 levels ≥ 3000 pg/mL^29^. Intra-amniotic infection was defined as microbial invasion of the amniotic cavity and intra-amniotic inflammation^24^, while intra-amniotic inflammation in the absence of microbial invasion of the amniotic cavity led to a diagnosis of sterile intra-amniotic inflammation^24^. Colonization of the amniotic cavity was defined as microbial invasion of the amniotic cavity with no intra-amniotic inflammation. Cases with neither microbial invasion of the amniotic cavity nor intra-amniotic inflammation were designated negative amniotic fluid^24^.

### Quantification of progranulin in cervical and amniotic fluid

Progranulin levels were assessed in the cervical and amniotic fluid samples. An enzyme-linked immunosorbent assay (ELISA) and the Human Progranulin ELISA Kit (BioVendor R&D, Brno, Czech Republic) were used for this analysis, according to the manufacturer’s instructions. Kit sensitivity was 18 pg/mL. The absorbance was read at 450 nm using a Multiskan RC ELISA reader (Thermo Fisher Scientific, Waltham, MA, USA). The inter- and intra-assay coefficients of variability were less than 8.0% and 4.4%, respectively.

### Statistics

The nonparametric Kruskal-Wallis test was applied for comparing the continuous variables (presented as median values; interquartile range [IQR]) among the demographic and clinical characteristics. The corresponding categorical variables, presented as numbers (%), were compared using Fisher’s exact test. Normality of data was tested with the Anderson–Darling test. The nonparametric Kruskal–Wallis or the Mann–Whitney *U* test was performed, as appropriate, for statistical analyses, since amniotic and cervical fluid levels were not normally distributed; these results are presented as median values (IQR). Spearman’s partial correlation analysis was performed to adjust for gestational age at sampling, while the relationship between the respective progranulin levels in amniotic and cervical fluids was assessed with Spearman’s correlation. All *p* values were obtained using two-tailed tests and differences were regarded as statistically significant at *p* < 0.05. All statistical analyses were performed using GraphPad Prism, version 8.1.1. for Mac OS X (GraphPad Software, San Diego, CA, USA) or the Statistical Package for Social Sciences (SPSS), version 19.0 for Mac OS X (SPSS Inc., Chicago, IL, USA).

## Electronic supplementary material

Below is the link to the electronic supplementary material.

Supplementary Material 1

## Data Availability

The data available is a part of the manuscript in the supplementary file.
